# Supplementary material for: Neuron-specific repression of alternative splicing by the conserved CELF protein UNC-75 in Caenorhabditis elegans
Source: Genetics. 2025 Mar 10;229(4):iyaf025. doi: 10.1093/genetics/iyaf025 (PMC12005262; doi:10.1093/genetics/iyaf025)
Supplement: iyaf025_Supplementary_Data [file iyaf025_supplementary_data.zip › Figure_S1_GENETICS-2024-307490.pdf]

Domain	Sequence	Alignment
RRM1	***C. elegans* UNC-75**	KLFVGQIPRNLEEKDLRHLFEQFGKIYEFTILKDKYTGMHKGCAFLTYCHRDSAVRCQATLHDQKTLPGMNRAMQVK
	H. sapiens CELF3	KLFVGQIPRHLEEKDLKPIFEQFGRIFELTVIKDKYTGLHKGCAFLTYCARDSALKAQSALHEQKTLPGMNRPIQVK
	H. sapiens CELF4	KLFIGQIPRNLDEKDLKPLFEEFGKIYELTVLKDRFTGMHKGCAFLTYCERESALKAQSALHEQKTLPGMNRPIQVK
	H. sapiens CELF5	KLFVGQIPRHLDEKDLKPLFEQFGRIYELTVLKDPYTGMHKGCAFLTYCARDSAIKAQTALHEQKTLPGMARPIQVK
	H. sapiens CELF6	KLFVGQIPRGLDEQDLKPLFEEFGRIYELTVLKDRLTGIHKGCAFLTYCARDSALKAQSALHEQKTLPGMNRPIQVK
RRM2	***C. elegans* UNC-75**	KLFIGMLSKQQSEDEVRALFATFGELDEVTVLRGADGASKGCAFVKYKHGLDAHMAISALHGSQTMPGASSSLVVK
	H. sapiens CELF3	KLFVGMLGKQQTDEDVRKMFEPFGTIDECTVLRGPDGISKGCAFVKFQTHAEAQAAINTLHSSRTLPGASSSLVVK
	H. sapiens CELF4	KLFVGMLNKQQSEDDVRRLFEAFGNIEECTILRGPDGNSKGCAFVKYSSHAEAQAAINALHGSQTMPGASSSLVVK
	H. sapiens CELF5	KLFVGMLNKQQSEEDVLRLFQPFGVIDECTVLRGPDGSSKGCAFVKFSSHIEAQAAIHALHGSQTMPGASSSLVVK
	H. sapiens CELF6	KLFVGMLGKQQGEEDVRRLFQPFGHIEECTVLRSPDGISKGCAFVKFGSQGEAQAAIRGLHGSRTMAGASSSLVVK
RRM3	***C. elegans* UNC-75**	NLFIYHLPQEFGDAELIQMFAPFGHIVSAKVFVDRATNQSKCFGFVSYDNIHSSQAAITAMNGFQIGMKRLKVQ
	H. sapiens CELF3	NIFIYHLPQEFTDSEIIQMFVPFGHVISAKVFVDRATNQSKCFGFVSFDNPASAQAAIQAMNGFQIGMKRLKVQ
	H. sapiens CELF4	NLFIYHLPQEFGDAELMQMFLPFG--------------------FVSFDNPASAQTAIQAMNGFQIGMKRLKVQ
	H. sapiens CELF5	NLFIYHLPQEFGDTEITQMFLPFGNIISSKVFMDRATNQSKCFGFVSFDNPASAQAAIQAMNGFQIGMKRLKVQ
	H. sapiens CELF6	NLFIYHLPQEFGDAELIQTFLPFGAVVSAKVFVDRATNQSKCFGFVSFDNPTSAQTAIQAMNGFQIGMKRLKVQ

Figure S1: Conservation of UNC-75 RNA Recognition motifs with human CELF proteins
Multiple sequence alignment of all three RRM domains of UNC-75, and human CELF3, CELF4, CELF5, and CELF6. Highly conserved regions are indicated in blue (darker blue = invariant regions, lighter blue = 1-2 changes at aligned amino acid position), and regions not conserved are indicated in white.
