# Supplementary material for: Neuron-specific repression of alternative splicing by the conserved CELF protein UNC-75 in Caenorhabditis elegans
Source: Genetics. 2025 Mar 10;229(4):iyaf025. doi: 10.1093/genetics/iyaf025 (PMC12005262; doi:10.1093/genetics/iyaf025)
Supplement: iyaf025_Supplementary_Data [file iyaf025_supplementary_data.zip › Figure_S2_GENETICS-2024-307490.pdf]

Figure S2

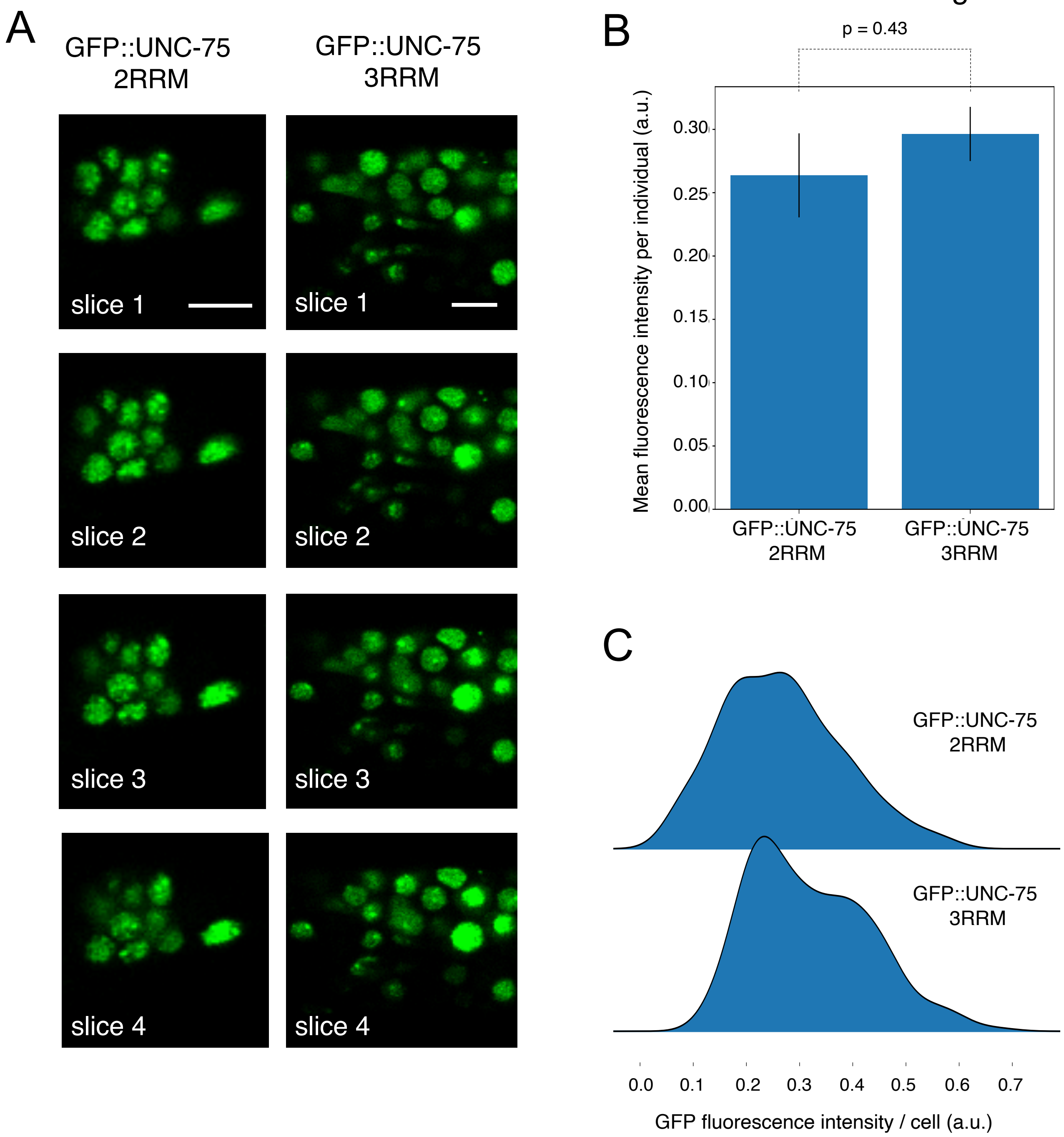

Figure S2: GFP::UNC-75 2RRM and 3RRM fusion protein isoforms are expressed at similar levels and localize to nuclear foci.

A) Fluorescence microscopy images showing GFP signal from representative neuronal nuclei from animals expressing GFP::UNC-75 2RRM (left column images) or GFP::UNC-75 3RRM (right column images). Rows represent snapshots of four consecutive Z slices. Nuclear foci are observable within some of the nuclei. **B)** Bar plots showing mean intensity +/- 1 standard deviation for GFP::UNC-75 2RRM and 3RRM isoforms. p-values are calculated from a T--test **C)** Ridgeline plots display distribution of intensity values used to derive mean values in (B). For both (B) and (C), for each GFP fusion protein, 5 individual animals were imaged from each transgenic strain. In (B) the mean signal from all cells sampled in an individual animal are presented, +/- 1 S.E.M. In (C), the distribution of signals from individual cells across all individuals for a given transgenic strain are displayed (~200 cells / strain).
