# Supplementary material for: Neuron-specific repression of alternative splicing by the conserved CELF protein UNC-75 in Caenorhabditis elegans
Source: Genetics. 2025 Mar 10;229(4):iyaf025. doi: 10.1093/genetics/iyaf025 (PMC12005262; doi:10.1093/genetics/iyaf025)
Supplement: iyaf025_Supplementary_Data [file iyaf025_supplementary_data.zip › Figure_S3_GENETICS-2024-307490.pdf]

Figure S3

```
C. zanz     ------------------------mrATIPPTVAVvaPPTAAvMGQqGQssHdMlRASST  SSTDSNGFPVKDPDAIKLFVGQIPRNLEEKDLRHLFEQFGKIYEFTILKDKYTGMHKGCA
C. Sp. 54   ------------mvhrrrLlarqkgLvTIPPTVAlAPPPsAAe---MGQQQHEM-RASST  SSTDSNGFPVKDPDAIKLFVGQIPRNLEEKDLRHLFEQFGKIYEFTILKDKYTGMHKGCA
C. elegans  ----------------------------------------------MGQQQHEM-RASST  SSTDSNGFPVKDPDAIKLFVGQIPRNLEEKDLRHLFEQFGKIYEFTILKDKYTGMHKGCA
C. inop     ----------------------------------------------MGQQQHdv-RASST  SSTDSNGFPVKDPDAIKLFVGQIPRNLEEKDLRHLFEQFGKIYEFTILKDKYTGMHKGCA
C. kama     ------------------------------------------------------------  ------------------------------------------------------------
C. oiwi     ----------------------------------------------MGQpQHEM-RASST  SSTDSNGFPVKDPDAIKLFVGQIPRNLEEKDLRHLFEQFGKIYEFTILKDKYTGMHKGCA
C. trop     ------------------------------------------------------------  ------------------------------------------------------------
C. late     maslsrfaslqrrhrsgsfldifelLATIPPTVAIAPPPTAAgMGQqGQQQHEM-RASST  SSTDSNGFPVKDPDAIKLFVGQIPRNLEEKDLRHLFEQFGKIYEFTILKDKYTGMHKGCA
C. Sp 33    ----------------------------------------------MGQQgHEM-RASST  SSTDSNGFPVKDPDAIKLFVGQIPRNLEEKDLRHLFEQFGKIYEFTILKDKYTGMHKGCA
C. trib     ------------------MhlnivkrATIPPTVAVvaPPTAAvMGQqGQ-sHEM-RASST  SSTDSNGFPVKDPDAIKLFVGQIPRNLEEKDLRHLFEQFGKIYEFTILKDKYTGMHKGCA
C. brig     ----------------------------------------------MvQQgHEM-RASST  SSTDSNGFPVKDaDAIKLFVGQIPRNLEEKDLRHLFEQFGKIYEFTILKDKYTGMHKGCA
C. nigo     ----------------------------------------------MvQQgHEM-RASST  SSTDSNGFPVKDaDAIKLFVGQIPRNLEEKDLRHLFEQFGKIYEFTILKDKYTGMHKGCA
C. doug     ----------------------------------------------MGQQgHEM-RASST  SSTDSNGFPVKDPDAIKLFVGQIPRNLEEKDLRHLFEQFGKIYEFTILKDKYTGMHKGCA
C. bren     ------------------------------------------------------------  ------------------------------------------------------------
C. Sp 48    --------------maslLfteteiLATIPPTVAVAPqPsAAgMGQqGQpQHEM-RASST  SSTDSNGFPVKDPDAIKLFVGQIPRNLEEKDLRHLFEQFGKIYEFTILKDKYTGMHKGCA
```

```
C. zanz     FLTFCHRDSAQRCQTaLHDQKTLPG----------------MNRAMQVKPADTDSRPASP  KDKtDDKKLFIGMLSKQQSEDDVRTLFAAFGELDEVTVLRGADGASKGCAFVKYKSGFDA
C. Sp. 54   FLTyCHRDSAlRCQTTLHDQKTLPGtqsqkqyfsaeFpEfFMNRAMQVKPADTDSRPASP  KDKMDDKKLFIGMLSKQQSEDDVRaLFstFGaLDEVTVLRGADGASKGCAFVKYKdGiDA
C. elegans  FLTyCHRDSAvRCQaTLHDQKTLPG----------------MNRAMQVKPADTDSRPASP  KDKMDDKKLFIGMLSKQQSEDeVRaLFAtFGELDEVTVLRGADGASKGCAFVKYKhGlDA
C. inop     FLTyCHReSAiRCQTTLHDQKTLPG----------------MNRAMQVKPADTDSRPASP  KDKMDDKKLFIGMLSKQQSEDDVReLFAtFGELDEVTVLRGADGASKGCAFVKYKhGlDA
C. kama     -----------------------------------------MNRAMQVKPADTDSRPASP  KDKMDDKKLFIGMLSKQQtEDDVRnLFstFGELeEVTVLRGADGASKGCAFVKYKhGlDA
C. oiwi     FLTyCHRDSAvRCQTTLHDQKTLPGv--------phFlKsWMNRAMQVKPADTDSRPASP  KDKMDDKKLFIGMLSKQQSEDDVRnLFstFGELeEVTVLRGADGASKGCAFVKYKhGlDA
C. trop     ---------------------------------------------MQVKPADTDSRPASP  KDKMDDKKLFIGMLSKtQSEDDVRTLFAAFGELDEVTVLRGADGASKGCAFVKYKSGFDA
C. late     FLTFCHRDSAQRCQTTLHDQKTLPG------------------------PADTDSRPASP  KDKaDDKKLFIGMLSKQQSEDDVRTLFAAFGELDEVTVLRGADGASKGCAFVKYKSGFDA
C. Sp 33    FLTFCHRDSAQRCQTTLHDQKTLPG----------------MNRAMQVKPADTDSRPASP  KDKaDDKKLFIGMLSKQQSEDDVRTLFAAFGELeEVTVLRGADGASKGCAFVKYKSGFDA
C. trib     FLTFCHRDSAQRCQTaLHDQKTLPG----------------MNRAMQVKPADTDSRPASP  KDKaDDKKLFIGMLSKQQSEDDVRvLFAAFGELDEVTVLRGADGASKGCAFVKYKSGFDA
C. brig     FLTFCHRDSAQRCQTTLHDQKTLPG----------------MNRAMQVKPADTDSRPASP  KDKMDDKKLFIGMLSKQQSEDDVRTLFAAFGELDEVTVLRGADGASKGCAFVKYKSGFDA
C. nigo     FLTFCHRDSAQRCQTTLHDQKTLPG----------------MNRAMQVKPADTDSRPASP  KDKMDDKKLFIGMLSKQQSEDDVRTLFAAFGELDEVTVLRGADGASKGCAFVKYKSGFDA
C. doug     FLTyCHRDSAQRCQTaLHDQKTLPG----------------MNRAMQVKPADTDSRPASP  KDKMDDKKLFIGMLSKtQSEDDVRTLFAAFGELDEVTVLRGADGASKGCAFVKYKSGFDA
C. bren     -----------------------------------------MNRAMQVKPADTDSRPASP  KDKMDDKKLFIGMLSKtQSEDDVRTLFAAFGELDEVTVLRGADGvSKGCAFVKYKSGFDA
C. Sp 48    FLTyCHRDSAtRCQTTLHDQKTLPG----------------MNRAMQVKPADTDSRPASP  KDKMDDKKLFIGMLSKtQSEDDVRTLFAAFGELDEVTVLRGADGvSKGCAFVKYKSGFDA
```

```
C. zanz     HMAISALHGSQTMPGASSSLVVKYADTEkERQNRRMQQMAAQMGMLNPmLaNQVGLQYN-  AY-QQVLQQQAm-vAQTSAASVAYLPLLQQQh-vAAaDPLQMLQLQAAA------AAA--
C. Sp. 54   HMAISALHGSQTMPGASSSLVVKYADTERERQNRRMQQMAAQMGMLNPmLINQVGLQYN-  AY-QQVLQQQAI-AAQTSAASVAYLPLLQQQS-SAATDPLQMLQLQAAAA-----AAASA
C. elegans  HMAISALHGSQTMPGASSSLVVKYADTEkERQNRRMQQMAAQMGMLNPmLVNQVGmQYN-  AY-QQVLQQQslAAqtnamASaAYLPLLQQQT----TDPLhvLQLQAAAA-----AAq--
C. inop     HMAISALHGSQTMPGASSSLVVKYADTERERQNRRMQQMAAQMGMLNPmLVNQVGLQYN-  AY-QQVLQQQAlAAqtsavASaAYLPLLQQQT-SAAsDPLhMLQLQAA-------AAA--
C. kama     HMAISALHGSQTMPGASSSLVVKYADTERERQNRRMQQMAAQMGMLNPmLINQVGLQYN-  AY-QtaLQQQAlAAqtsaAASVAYLPLLQQpS-SAATDPLQMLQLQAAAA-----AAA--
C. oiwi     HMAISALHGSQTMPGASSSLVVKYADTERERQNRRMQQMAAQMGMLNPmLINQVGLQYs-  AY-QtaLQQQAlAAqtsaAASVAYLPLLQQpS-SAATDPLQMLQLQAAAA-----AAA--
C. trop     HMAISALHGSQTMPGASSSLVVKYADTERERQNRRMQQMAAQMGMLNPLLVNQVGLQYNA  AY-QQVLQhQAI-AAQTSAASVAYLPLLQQQT--sATDPLQMLQfQA--------AAA--
C. late     HMAISALHGSQTMPGASSSLVVKYADTEkERQNRRMQQMAAQMGMLNPLLVNQVGLQYN-  AYAQQVLQQQtI-AAhaSAASVAYLPLLQQQqAaAATDPLQMLQLQAAA------AAA--
C. Sp 33    HMAISALHGSQTMPGASSSLVVKYADTEkERQNRRMQQMAAQMGMLNPLLVNQVGLQYN-  AYAQQVLQQQtI-AAhaSAASVAYLPLLQQQqAaAATDPLQMLQLQAAA------AAAAS
C. trib     HMAISALHGSQTMPGASSSLVVKYADTEkERQNRRMQQMAAQMGMLNPmLaNQVGLQYN-  AY-QQVLQQQAm-vAQTSAASVAYLPLLQQQh-vAAaDsLQMLQLQAAA------AAA--
C. brig     HMAISALHGSQTMPGASSSLVVKYADTEkERQNRRMQQMAAQMGMLNPLLVNQVGLQYN-  tY-QQVLQQQAI-AAQTSAASVAYLPLLQQQh--tATDPLQMLQfQAA-------AAA--
C. nigo     HMAISALHGSQTMPGASSSLVVKYADTEkERQNRRMQQMAAQMGMLNPLLVNQVGLQYN-  tY-QQVLQQQAI-AAQTSAASVAYLPLLQQQh--tATDPLQMLQfQAA-------AAA--
C. doug     HMAISALHGSQTMPGASSSLVVKYADTERERQNRRMQQMAAQMGMLNPLLVNQVGLQYN-  AY-QQVLQQQAI-AAQTSAASVAYLPLLQQQp--ttTDPLQMLQfQAA-------AAA--
C. bren     HMAISALHGSQTMPGASSSLVVKYADTERERQNRRMQQMAAQMGMLNPLLVNQVGLQYNA  AY-QhVLQhQtlAAAQTSAASmAYLPLLQQQT-SAAaDPLQMLQfQAAAAAAASaAAA--
C. Sp 48    HMAISALHGSQTMPGASSSLVVKYADTERERQNRRMQQMAAQMGMLNPLLVNQVGLQYNA  AY-QhVLQhQtlAAAQTSAASmAYLPLLQQQT-SAtaDPLQMLQfQAAAAAAAS-AAA--
```

```
C. zanz     AAAnPVMq-------pQpPQLAAQLQGLQS-------AAAVsQNQHYALAAQ-LAQQQAA  QQAQQAAVAAHa----qQQaAQSHQQVHP-VSQsTAsTtSSQsDvanSYAAAAAAAAAAA
C. Sp. 54   AAsTPVMP------QQpQqQLAAQL-GLQt--------AqaAQNQHYALAAQALAQQQAA  QQAQvqAqAAa------aQavshqQQVHP-VSQaqStTSqS---DTStY--glAAAAAAA
C. elegans  AAAnPVLsqqtqqqQQQQqQLAAQL-qLQS---------AaAQNpHYALAAQALAQQQAA  QQAQ--AVvAH-------shAQvHQhqtaqVtsTaShaqTenp-aaSSY-gslAAAAAAA
C. inop     AAAnPVLP--------QQPQLAAQL-GLQthaaaaaaAAAaAQNpHYALAAQALAQQQAA  aaqQaqAVvAH------sahSQvHQQpHsAshtTTStaShSQnDtTSSY--glvAAAAAA
C. kama     AsAaPVLP--------QQPQLAAQL-GLQS--------AAaAQNpHYALAAQALAQQQAA  QQ------------------------HPSVhpvsqATSTSsqsDTSSY--nlAAAAAAA
C. oiwi     AsAaPVLP--------QQPQLAAQL-GLQS--------AAaAQNpHYALAAQALAQQQAA  QQAaQ---------------------HPSVhpvsqATSTSsqsDTSSY--nlAAAAAAA
C. trop     AAAaPVLq--------QQPQqlAaLQGLQS--------AAVAQNQHYALAAQALAQQQAA  QQAQ--vV---------hpphQgHQQVHP-VSQpTStqS-----DTSSY-AAAAAAAAAA
C. late     AAsTPsiP-------QQpqQLAAQLQGLQS-----------AQNQHYALAAQALAQQQqA  va---------------------HQQVHP-VSQsTSsTaT----DTtSY--glAAAAAAA
C. Sp 33    AAATPsiP--------QQPQL-AQLQGLQS--------AAaAQNQHYALAAQALAQQQAA  QQAQ--AVAAH-----------SHQQVHP-VSQsTgsTtTA---DTttY--glAAAAAAA
C. trib     AAATPVMq-------pQpPQL-AQLQGLQS-------AAAVsQNpHYALAAQALAQQQAA  QQAQhAAVAAH------aQqqQSHQQVHP-VSQssAsTtSSQsDaanSYAAAAAAAAAAA
C. brig     AAATPVMP-------QQQPQLAAQiQGLQS-------AAAVsQNpHYALAAQALAQQQAA  QQAQQAAVAAH-----AQhtAagHQQVHsgVnQTapATSTAQgDaaSSYAAAAAAAAAAA
C. nigo     AAATPVMP-------QQQPQLAAQiQGLQS-------AAAVsQNpHYALAAQALAQQQAA  QQAQQAAVAAH-----AQhtAagHQQVHsgVSQTTpATSTAQgDaaSSYAAAAAAAAAAA
C. doug     AAATPVMP-------QQnPQLAAQL-GLQt--------AAVAQNQHYALAAQALAQQQAA  aaqQaqvVAAHQAQQhSQpphpgHQQVHPAVqQaTAAsaqpt--DTSSYAAAAAAAAAAA
C. bren     sAAmPVpP-------QQpqQLAAQfQGLQS--------AAVAQNQHYALAAQALAQQQAA  QQAQ--vVAAHQAQQ-tQQahQSHQpVHP-VnQTTpAapppn--DTSSY--AAAAAAAAA
C. Sp 48    sAAmPVpq-------QQptQLAAQfQGLQS--------AAVAQNQHYALAAQALAQQQAA  QQAQ--vVAAHQvQQ-tQQahQSHQpVHP-VnQTTpAapppn--DsSSY--AAAAAAAAA
```

```
C. zanz     NYNSLitGiE-SQH-----------------NAAAALQLAQMQQQAAAALPMiTPkEasp  irhpmiPwsiqkhviggrhqqqhhyntknPyflarnsKteFlrRgqrpkcTtlqrCvk
C. Sp. 54   NsySLLtGMd-pQHksefhypnstystiytAdAAAALQLAQiQQQAAAALPMVTPREV--  ----LGPDGCNLFIYHLPQEFGDAELIQMFAPFGHVVSAKV-FVDR-----ATNQSKCFG
C. elegans  NYsSLLSGME-SQH-----------------NAAAALQLAQiQQQAAAALPMVTPREV--  ----LGPDGCNLFIYHLPQEFGDAELIQMFAPFGHIVSAKV-FVDR-----ATNQSKCFG
C. inop     NYNSLLSGME-tQH-----------------NAAAALQLAQiQQQAAAALPMVTPREV--  ----LGPDGCNLFIYHLPQEFGDAELIQMFAPFGHVVSAKV-FVDR-----ATNQSKCFG
C. kama     NYsnLLtGidTSQH-----------------NAvAALQLAQMQQQAAAALPMVTPkEV--  ----LGPDGCNLFIYHLPQEFGDAELIQMFAPFGHVVSAKV-FVDR-----ATNQSKCFG
C. oiwi     NYsnLLtGidTSQH-----------------NAvAALQLAQMQQQAAAALPMVTPkEV--  ----LGPDGCNLFIYHLPQEFGDAELIQMFAPFGHVVSAKV-FVDR-----ATNQSKCFG
C. trop     NYNSLLSGME-SQH-----------------NAAAALQLAQiQQQAAAALPlVTPRE---  ------------------------------getsyrISA---------------------
C. late     NYtSLLSGME-SQH-----------------NAAAALQLAQMQQQAAAALPMVTPREV--  ----LGPDGCNLFIYHLPQEFGDAELIQMFAPFGHVVSAKV-FVDR-----ATNQSKCFG
C. Sp 33    NYsSLLSGME-SQH-----------------NAAAALQLAQMQQQAAAALPMVTPREg--  ------------FvsydnihssqAaiaaMngfq---------------------------
C. trib     sYNSLitGiE-SQH---------------iAdAAAALQLAQMQhQAAAALPMiTPkEV--  ----LGPDGCNLFIYHLPQEFGDAELIQMFAPFGHVVSAKV-FVDR-----ATNQSKCFG
C. brig     sYNSLiSGiE-SQH-----------------NAAAALQLAQMQQQAAAALPMiTPkEV--  ----LGPDGCNLFIYHLPQEFGDAELIQMFAPFGHVVSAKV-FVDR-----ATNQSKCFG
C. nigo     sYNSLiSGiE-SQH-----------------NAAAALQLAQMQQQAAAALPMiTPkEV--  ----LGPDGCNLFIYHLPQEFGDAELIQMFAPFGHVVSAKV-FVDR-----ATNQSKCFG
C. doug     NYNSiLSGME-SQH-----------------NAAAALQLAQMQQhAAAALPMVTPREV--  ----LGPDGCNLFIYHLPQEFGDAELIQMFAPFGHVVSAKV-FVDR-----ATNQSKCFG
C. bren     NYNSiLSGME-SQH-----------------NAAAALQLAQiQQQAAAALPMVTPREV--  ----LGPDGCNLFIYHLPQEFGDAELIQMFAPFGHVVSAKV-FVDR-----ATNQSKCFv
C. Sp 48    NYNSiLSGME-SQH-----------------NAAAALQLAQiQQQAAAALPMVTPREV--  ----LGPDGCNLFIYHLPQEFGDAELIQMFAPFGHVVSAKV-FVDR-----ATNQSKCFG
```

```
C. zanz     wcldeleildripkcfqhrcwcieqdsplnapmsyfttteepIlAvestveGsgdsedit  enPkqEp--------------IqfdLieyLkNwffespsY
C. Sp. 54   scfe--------------------------------------------FffGyili----  ----------------------------------------
C. elegans  FVSYD-----------------------------NIHSSQAAItAMNGFQIGMKRLKVQL  KRPRnES------------------------------RPY
C. inop     FVSYD-----------------------------NIHSSQAAIAAMNGFQIGMKRLKVQL  KRPRnES------------------------------RPY
C. kama     FVSYD-----------------------------NmHStQAAIAAMNGFQIGMKRLKVQL  KRPRnES------------------------------RPY
C. oiwi     FVSYD-----------------------------NmHStQAAIAAMNGFQIGMKRLKVQL  KRPRnESpaartssrrhlrppMmllVkrpLaNhhesvRgf
C. trop     ------------------------------------------------------------  ----------------------------------------
C. late     scfei-------------------------------------------Ffl---------  ----------------------------------------
C. Sp 33    --------------------------------------------------IGMKRLKVQL  KRPREsa------------------------------RPY
C. trib     FVSYD-----------------------------NIHSSQAAIAAMNGFQIGMKRLKVQL  KRPREsS------------------------------RPY
C. brig     FVSYD-----------------------------NIHSSQAAIAAMNGFQIGMKRLKVQL  KRPREaS------------------------------RPY
C. nigo     FVSYD-----------------------------NIHSSQAAIAAMNGlQIGMKRLKVQL  KRPREaS------------------------------RPY
C. doug     FVSYD-----------------------------NIHSSQAAIAAMNGFQIGMKRLKVQL  KRPREsS------------------------------RPY
C. bren     ------------------------------------------------------------  ----------------------------------------
C. Sp 48    FVSYD-----------------------------NIHSSQAAIAAMNGFQIGMKRLKVQL  KRPREsS------------------------------RPY
```

Figure S3: Genes encoding UNC-75 protein orthologs are deeply conserved across Caenorhabditis species
unc-75 ortholog sequence alignments obtained from 15 *Caenorhabditis* species which are part of the *elegans* super group. Amino acid positions that are identical across all species are highlighted in teal, while amino acids showing conservation across most species are highlighted in gray. Species abbreviations: *kama = kamaaina; oiwi = oiwi; trop = tropicalis; bren = brenneri; inop = inopinata; doug = doughertyi; late = latens; brig = briggsae; nigo – nigoni; trib = tribulationis; zanz = Zanzibari; eleg = elegans.*
