# Supplementary material for: Neuron-specific repression of alternative splicing by the conserved CELF protein UNC-75 in Caenorhabditis elegans
Source: Genetics. 2025 Mar 10;229(4):iyaf025. doi: 10.1093/genetics/iyaf025 (PMC12005262; doi:10.1093/genetics/iyaf025)
Supplement: iyaf025_Supplementary_Data [file iyaf025_supplementary_data.zip › Figure_S4_GENETICS-2024-307490.pdf]

Figure S4

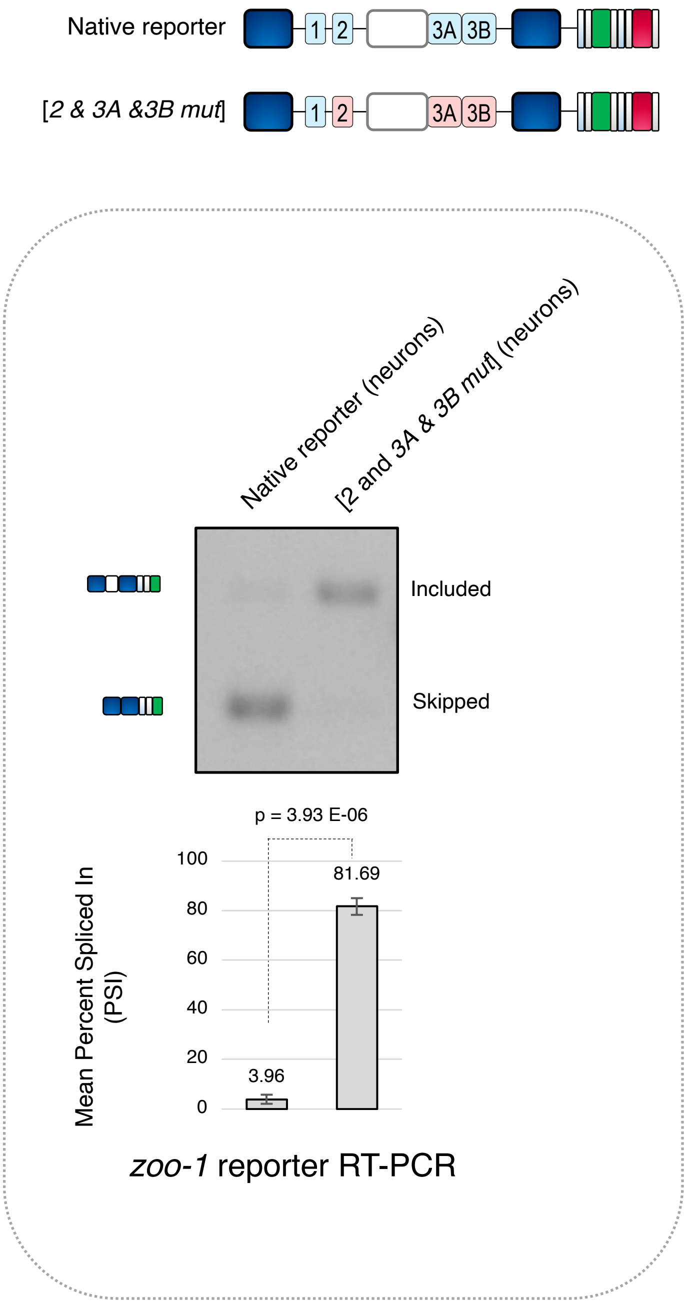

Figure S4: Combined disruption of regions 2, 3A and 3B leads to robust *zoo-1* exon 9 inclusion in neurons, recapitulating a loss of UNC-75 protein.
Top panel: schematics of mutagenesis experiments disrupting two UNC-75 consensus sequences immediately adjacent to the 5′ splice site flanking exon 9 and region 2 upstream of exon 9. Pink labeled boxes denote regions mutated as shown in Figure 3. Bottom: representative RT-PCR and densitometric measurements assessing *zoo-1* exon splicing patterns from the native reporter (left) and the 2 & 3A & 3B mutant reporter (right) in neurons. n = 3 replicates for each data point. Mean PSI +/- 1 standard deviation is plotted, and p-values are calculated from Student's t-test.
