# Supplementary material for: Neuron-specific repression of alternative splicing by the conserved CELF protein UNC-75 in Caenorhabditis elegans
Source: Genetics. 2025 Mar 10;229(4):iyaf025. doi: 10.1093/genetics/iyaf025 (PMC12005262; doi:10.1093/genetics/iyaf025)
Supplement: iyaf025_Supplementary_Data [file iyaf025_supplementary_data.zip › Figure_S5_GENETICS-2024-307490.pdf]

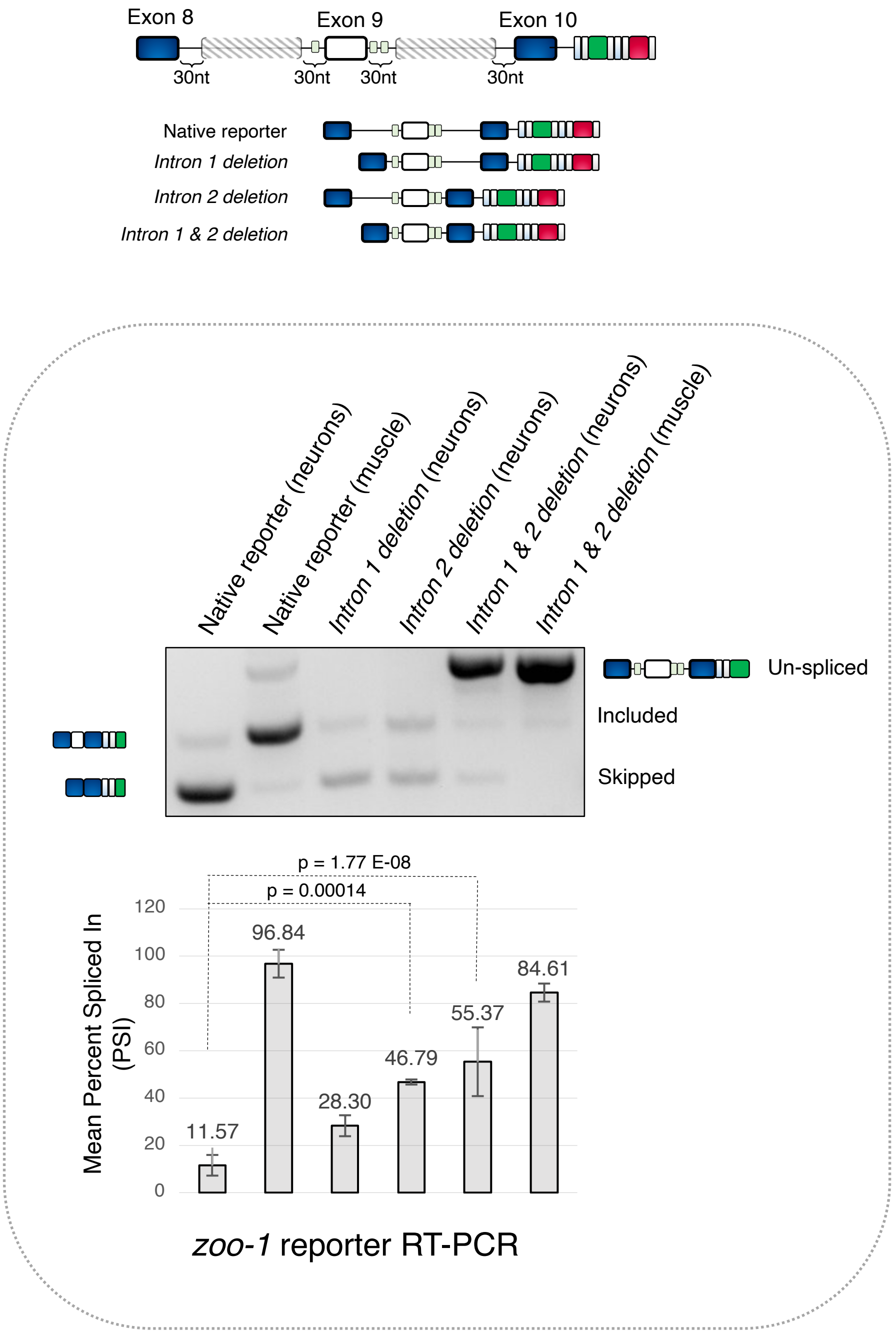

Figure S5: Deletion of large blocks of upstream and downstream intronic sequences flanking zoo-1 exon 9 impacts exon usage and overall splicing efficiency

Top panel: Schematic of *zoo-1* reporter intron deletion experiment, displaying the native reporter, reporters with large portions of each intron deleted, and a reporter with large portions of both upstream and downstream introns deleted. Bottom panels: Representative RT-PCR, and densitometric measurements of the *zoo-1* reporters described in the above schematic. n = 3 replicates for each data point. Mean PSI +/- 1 standard deviation is plotted, and p-values are calculated from Student's t-test.
